# Systemic neutrophil migration and rapid consumption of neutrophils in the spleen

**DOI:** 10.1016/j.dib.2018.08.090

**Published:** 2018-08-30

**Authors:** Yuji Takeda, Tomoyuki Kato, Nobuhito Nemoto, Akemi Araki, Mohammad Yeashin Gazi, Hidetoshi Nara, Hironobu Asao

**Affiliations:** a Department of Immunology, Yamagata University Faculty of Medicine, 2-2-2 Iida-Nishi, Yamagata 990-9585, Japan; b Department of Urology, Yamagata University Faculty of Medicine, 2-2-2 Iida-Nishi, Yamagata 990-9585, Japan; c Department of Orthopaedics, Yamagata University Faculty of Medicine, 2-2-2 Iida-Nishi, Yamagata 990-9585, Japan

## Abstract

The systemic migration of neutrophils is not fully understood. In this study, we purified neutrophils from rat peripheral blood and labeled them with [^51^Cr] sodium chromate. The labeled cells were injected into the tail veins of rats, and were traced. Neutrophils were rapidly trapped in the liver and the spleen within 6 h. The migration ratios of neutrophils in the lung and the gut were lower compared with those in the liver and the spleen. Interestingly, migrated cells into the spleen were rapidly phagocytosed by monocytes/macrophages. Therefore, accumulation of intact neutrophils in the spleen may be difficult to measure.

**Specifications Table**

**Table:** 

Subject area	*Biology*
More specific subject area	*Immunology*
Type of data	*Figure*
How data was acquired	*Γ-counter*
Data format	*Analyzed*
Experimental factors	*Rat and radio-labeled peripheral neutrophils*
Experimental features	*The systemic neutrophil migration was observed in rat. Phagocytosed neutrophils were separated by Ficoll gradient.*
Data source location	*Yamagata, Japan*
Data accessibility	*Article*

**Value of the data**

• Neutrophil distribution is important in various inflammatory diseases.
• Our data showed rapid neutrophil migration into various organs, and neutrophil clearance in the spleen under normal condition.
• These observations are useful for determining neutrophil accumulation and turnover in each organ.

## 1 Data

**Fig. 1:**
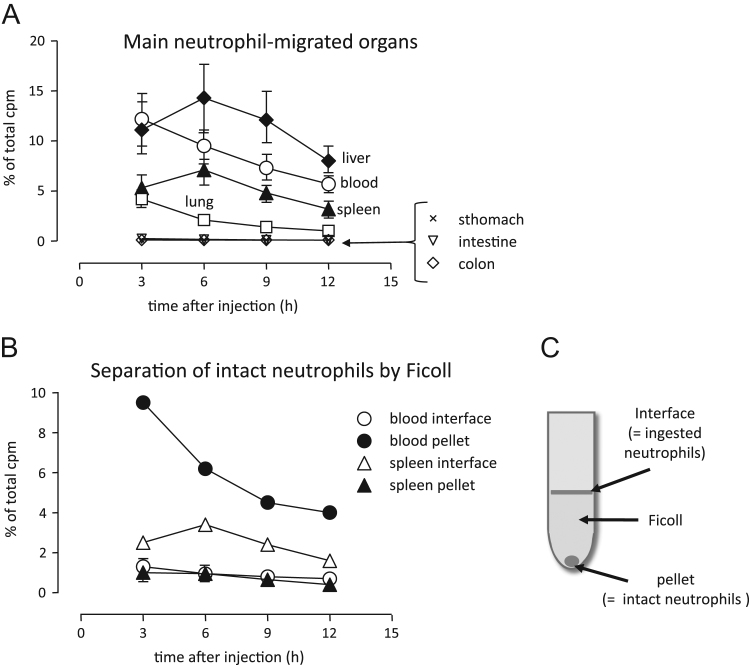
Systemic neutrophil migration and rapid consumption of neutrophils in the spleen. Neutrophils were purified from the peripheral blood of rats. The cells were labeled by sodium chromate-^51^Cr and injected intravenously through the tail vein. After the injection, rats were sacrificed, and various organs were harvested. The organs (A) or Ficoll-separated blood and spleen fractions (B) were measured by γ-counter. The percentage of counts per minute (cpm) was calculated using the following formula: % of total cpm = (cpm of each organ or the separated fraction) / (cpm of injected neutrophils) × 100. The data are shown as mean ± SE (*n* = 3).

The ^51^Cr -labeled neutrophils rapidly migrated into the spleen and the liver within 6 h (Fig.1A). The migration ratio in the gut was the lowest among all tested organs. It is well known that macrophages ingest pre-apoptotic neutrophils at a rapid rate. Thus, neutrophils ingested by macrophages (interface) and intact neutrophils (pellet) were separated by density gradient centrifugation, as shown in the left side of the scheme (Fig. 1B). It was observed that monocytes/macrophages remained at the interface of the Ficoll-Paque (1.077 g/mL), and the neutrophils precipitated to the bottom of the tubes following centrifugation (800 ×*g* for 10 min) (Fig. 1C). These data indicated that cells migrated into the spleen were rapidly phagocytosed by monocytes/macrophages.

## 2 Experimental design, materials and methods

Neutrophils were purified from peripheral blood of Wister-King-Aptakemann/Hkm (WKA/Hkm) rats (male; 5 to 6 weeks old; Funabashi Agriculture, Shizuoka, Japan), as described in our previous report [1]. The cells were labeled with [^51^Cr] sodium chromate (7.4 MBq/10^8^ cells; DAIICHI FINE CHEMICAL CO. LTD, Tokyo, Japan) in a CO_2_ incubator for 1 h at 37 °C. Labeled cells were washed twice and suspended in RPMI 1640 medium containing 10% FBS; 6 × 10^6^ cells were intravenously injected through the tail vein. Following injections, rats were sacrificed by hypovolemic shock with cardiac puncture under anesthesia, and various organs were harvested. The organs or Ficoll-separated blood and spleen fractions were measured by γ-counter (Pharmacia Biotech, Uppsala, Sweden) (Fig. 1).
